# Mucin Alterations in Response to High-Fat Diet and the Potential Protective Role of Chickpea Accessions

**DOI:** 10.3390/nu17193035

**Published:** 2025-09-23

**Authors:** Donatella Mentino, Daniela Semeraro, Nastasia Taldone, Stefano Pavan, Francesco Caponio, Patrizia Gena, Marianna Ranieri, Grazia Tamma, Marco Vito Guglielmi, Giovanni Scillitani, Stefania Fensore, Maria Mastrodonato

**Affiliations:** 1 Department of Biosciences, Biotechnology and Environment, University of Bari Aldo Moro, 70125 Bari, Italyn.taldone@alumni.uniba.it (N.T.); giovanni.scillitani@uniba.it (G.S.); maria.mastrodonato@uniba.it (M.M.); 2 Department of Soil, Plant, and Food Sciences, University of Bari Aldo Moro, 70125 Bari, Italy; 3 National Research Council of Italy (CNR)—Water Research Institute (IRSA), 74123 Taranto, Italy; 4 Department of Socio-Economic, Managerial and Statistical Studies, University of Chieti-Pescara, 65129 Pescara, Italy

**Keywords:** mucins, chickpeas, lectins, histochemical and immunohistochemical analysis

## Abstract

**Background/Objectives:** Unhealthy nutrition and lifestyles contribute to the development of chronic diseases such as cardiovascular disease, type 2 diabetes, and colorectal cancer. The Western diet can impair gastrointestinal motility and function. The underlying mechanisms that lead to changes in the mucus barrier and mucin profiles in response to these dietary patterns are still being studied. In mice, dietary fiber intake can improve the intestinal mucosal barrier function, enhance the differentiation process of goblet cells, and increase acidic mucin production. Our study aimed to investigate the effects of a high-fat diet (HFD) on colonic mucin expression and to assess whether chickpea accessions, known for their nutritional benefits, can mitigate these adverse effects. **Methods:** We investigated the effects of an HFD and an HFD associated with two accessions of chickpeas (HFD + MG_13; HFD + PI358934) on the mucin expression in murine colons of mice by conventional histochemistry and lectin-histochemistry, combined with chemical treatment and enzymatic digestion and immunohistochemistry. We evaluated possible alterations of Muc2, the main mucin secreted by the mucous cells of the colon. **Results:** HFD significantly reduced the expression of the mucin Muc2 and altered its composition in the colon. Compared to the CTRL group, distal and proximal measurements for HFD + PI, respectively, showed reductions of 78% and 36%; for the distal colon, a reduction of 34% was also observed for both the HFD and HFD + MG_13 diets. Changes in mucin glycosylation, including sialylation and sulfation, as well as residues such as *N*-acetylglucosamine, GalNAc, Mannose, and Galactose, were observed, suggesting a beneficial influence of chickpeas on mucosal integrity. In HFD + MG_13 these effects were reduced and resulted similar to the control. **Conclusions:** HFD reduces Muc2 expression in the colon and alters mucin composition: chickpea accessions, particularly MG_13, partially restore Muc2 levels and mucin oligosaccharide profiles, suggesting protective effects on the intestinal mucosal barrier.

## 1. Introduction

Lifestyle and dietary habits significantly influence human health. The Western diet, characterized by a high consumption of red and processed meats rich in saturated and trans fats, along with a substantial intake of added sugars, is associated with an increased risk of cardiovascular diseases, type 2 diabetes (T2D), inflammatory disorders, and colorectal cancer [1,2], which are key factors in metabolic syndrome [1]. Today, unhealthy lifestyle choices and poor dietary habits are becoming an increasingly widespread global problem, affecting children, adolescents, adults, and the elderly. Emerging evidence also suggests that high-fat diets (HFD) may contribute to the development of disorders such as anxiety, depression, and cognitive impairments [3]. Although an unbalanced diet can impair the motility and functionality of the gastrointestinal tract, the underlying mechanisms leading to changes in the mucus barrier and mucin profiles in response to such dietary patterns are still under investigation. The intestinal barrier, composed of a mucus layer secreted by goblet cells and the epithelial mucosa, serves as the primary defense against pathogens and harmful substances [4]. The main mucin involved in this process is MUC2, a heavily glycosylated glycoprotein that confers protective and barrier properties to the mucus layer [5]. MUC2’s absence in the colon allows bacteria to reach epithelial cells, invading crypts and enterocytes [6]. Mice deficient in Muc2 (the murine homolog of MUC2) spontaneously develop colitis, indicating that the protein is critical for colonic protection [6]. Alterations in mucin production or composition are associated with increased inflammatory susceptibility, as evidenced in murine models, which in turn may lead to conditions such as colitis and potentially promote carcinogenesis [6,7,8,9]. Despite these findings, limited data are available regarding the impact of HFD on mucin modulation, particularly in relation to functional foods such as legumes. Chickpeas (*Cicer arietinum* L.) may modulate mucin expression due to their fermentable fiber content, serving as a substrate for gut microbiota, as documented in Monk et al. (2017) [10,11]. Fermentation of this fiber leads to the production of short-chain fatty acids (SCFAs), including butyrate, which has demonstrated efficacy in enhancing gut barrier function [12]. The mechanism for this action involves stimulating mucin production (specifically Muc2), as detailed in the study of Van der Sluis et al. (2006) [6].

Chickpeas are rich in proteins, fibers, and bioactive compounds with demonstrated beneficial effects on metabolic and intestinal health [11,12]. Overall, they are a valuable harvest with numerous nutritional and health benefits [13]. *Cicer arietinum* L. is a widely cultivated pulse crop, especially in Afro-Asian countries, known for its high-quality protein and carbohydrate content [14]. They contain all essential amino acids except sulfur-containing ones, which can be complemented by cereals. The main storage carbohydrate is starch, along with dietary fiber, oligosaccharides, and simple sugars like glucose and sucrose. Chickpeas are low in lipids but rich in beneficial unsaturated fatty acids such as linoleic and oleic acids, and contain important sterols like β-sitosterol, campesterol, and stigmasterol. They provide essential minerals, including potassium, calcium, magnesium, and phosphorus, as well as vitamins like riboflavin, niacin, thiamin, folate, and β-carotene. Anti-nutritional factors present in chickpeas can be reduced through cooking. Although in vitro and in vivo studies have highlighted the potential protective properties of chickpeas, few investigations have specifically addressed how different chickpea accessions influence mucin expression and structure within the context of a high-fat dietary regimen. In this study, we aim to evaluate, in a murine model, the alterations in Muc2 expression and structural modifications induced by HFD supplemented with two distinct chickpea accessions: MG_13 and PI358934. The selection of these varieties is based on their similar content of protein, lipids, carbohydrates, and ash. However, the total phenolic content is lower in PI358934 than in MG_13. Using histochemical of lectins techniques, we seek to investigate whether these legumes exert modulatory effects on the mucosal barrier, thereby contributing to the understanding of their potential protective role against diet induced mucin alterations.

## 2. Materials and Methods

### 2.1. Animals

In agreement with Centrone et al. (2020) [15], forty-five C57BL/6J male mice (Envigo RMS S.R.L., San Pietro al Natisone, Udine, Italia) aged 3 weeks old and initially weighing about 18 g were kept in a Controlled Room Temperature at 25 °C, with a 12 h light/dark cycle. For a one-week acclimatization period, the mice were fed a control diet (CTRL) that was the same for all. According to the dietary formulation reported below, the mice had *ad libitum* access to the diet and water for 16 weeks. The individuals were divided into four groups of 12 each, as follows:

1. CTRL: standard diet with 10% fat, 24% protein, and 66% carbohydrates (3.514 kcal/kg);
2. HFD: diet with 45% fat, 19% protein, and 38% carbohydrates (4.496 kcal/kg);
3. HFD plus MG_13-chickpeas (HFD + MG_13): diet same as HFD (metabolized energy and composition), except that 10% of MG_13 raw crushed chickpea flour replaced crude fibers and ashes of HFD; details of diet are given in Costantini et al. (2021) [16];
4. HFD plus PI358934-chickpeas (HFD + PI): diet same as HFD (metabolized energy and composition), except that 10% of PI358934 raw crushed chickpea flour replaced crude fibers and ashes of HFD; details of diet are given in Costantini et al. (2021) [16]. In respect to MG_13, this diet has a similar content of protein, lipids, carbohydrates, and ash, and a lower phenolic content.

All experimental diets were stored at −20 °C. Body weight was monitored throughout the experiment. Mice were fasted for 12 h before sacrifice by cervical dislocation. The colon samples were removed, weighed, and quickly fixed for histochemical analysis. Animal experiments followed the Directive 2010/63/UE, enforced by Italian D.L. 26/2014, and approved by the animal care and the Committee of the University of Bari (OPBA), Bari, Italy, and the Italian Ministry of Health, Rome, Italy (authorization n.326/2018-PR).

### 2.2. Section Preparation

For each animal, two samples were collected: one immediately after the cecum (i.e., proximal colon) and the other located 2 cm from the anus (i.e., distal colon). The samples were immediately fixed in 10% neutral buffer formalin, dehydrated in a graduated series of ethanol, and then embedded in a hydrophobic wax paraffin. The sections were cut in series at 5 µm [17].

### 2.3. Classic Histochemical Staining

Histochemical techniques included Periodic Acid-Schiff (PAS) to highlight the presence of polysaccharides, glycoproteins, and other substances containing glycolic or aminohydroxyl groups in tissue samples. Acidic groups were identified by the high iron diamine–alcian blue at pH 2.5 (HID–AB 2.5) staining method, which simultaneously detects sulfated and nonsulfated acidic glycans. The staining procedure was carried out following the protocol described previously [18]. The sections were dewaxed, rehydrated, and then immersed for 18 h in a solution containing *N,N*-dimethylmetaphenylene diamine dihydrochloride, *N,N*-dimethylparaphenylene diamine dihydrochloride, and freshly prepared ferric chloride (60% w/vol). After washing with running tap water, sections were counterstained with 1% Alcian blue in 3% acetic acid (pH 2.5) for 30 min. Nuclei were counterstained with hematoxylin, then sections were dehydrated and mounted with DPX. Reagents were sourced from Sigma-Aldrich Inc. (St. Louis, MO, USA).

### 2.4. Lectins

To characterize oligosaccharides residues of glycoproteins were identified by lectin histochemistry. Sections were incubated with six different lectins conjugated with FITC (WGA, PNA, SBA, AAL, ConA, UEA-I) (Table 1) sourced from Vector Laboratories, Newark, CA, United States. The choice of lectins was based on literature data about the composition of oligosaccharide chains of mucins in the mucous cells [19,20,21]. The common names, sugar specificities, and concentrations of the lectins used are shown in Table 1. Sections were incubated for 1 h at RT with the FITC-lectin solution in their specific buffer, as indicated by manufacturers. Refer to Mentino et al. [17] and Mastrodonato et al. [22] for details. The controls implemented for lectin histochemistry included (1) substituting the lectin solution with buffer alone; (2) preincubating the tissue sections with the specific hapten sugar inhibitor at a concentration of 0.2 M; and (3) testing binding on samples known to contain mucins previously demonstrated to be labeled by the lectins used in this study, such as secretory epithelial tissues from the mouse duodenal villi and the intestines of triggerfish *Balistes capriscus* or pufferfish *Sphoeroides pachygaster* [22,23,24].

### 2.5. Enzymatic Treatments

The lectin histochemistry combined with enzymatic digestion by sialidase was used for indirect demonstration of the presence of terminal sialic acid in O-linked glycans and the identification of subterminal sugars to which it is bound. The link between FITC PNA and FITC SBA, with and without previous digestion with sialidase, was tested. Sialidase digestion was performed by incubating sections with 1 U/mL of sialidase extracted from *Clostridium perfrigens* (Neuraminidase Type V, Sigma-Aldrich) in 0.1 M acetate buffer pH 5.3, containing 10 mM CaCl2 for 48 h at 37 °C in a humid chamber [31]. The binding with FITC PNA and FITC SBA was performed by incubating the sections with a solution of HEPES 10 mM pH 7.5, containing lectin for 1 h at room temperature. The sections were subsequently rinsed in the same buffer and finally mounted with Fluoromount (Sigma). The desulfation reaction was carried out by a sequential methylation–saponification reaction. The sections were immersed in 0.15 N HCl in methanol (CHOH) for 5 h at 60 °C (methylation) and then rinsed in running water for 10 min and distilled water. They were then quickly washed in 70% alcohol and soaked in 0.5% potassium hydroxide (KOH) in 70% ethanol for 30 min at room temperature (saponification). The sections were rinsed again first in 70% alcohol, then in running water for 5 min and washed in distilled water [32]. Finally, the sections were incubated with lectin PNA and washed with the appropriate buffer. The slides were closed with Fluoromount.

### 2.6. Immunohistochemistry

Immunohistochemical assays were performed for Muc2. Protocols followed dealers’ specifications with minor modifications as follows. Before incubation with the corresponding antigen, sections underwent an antigen retrieval procedure [17] in 10 mM citrate-buffered saline pH 6.0 (CBS) for 20 min at 95 °C. The sections were then treated to reduce background interference with 0.3% Triton X-100 and 5% Normal Goat Serum (NGS) blocking buffer (Sigma) in Phosphate-Buffered Saline (PBS) for 30 min at 37 °C in a humid chamber. For Muc2 assay, the sections were incubated in a humid chamber overnight at 4 °C with rabbit polyclonal Muc2 (Invitrogen, Waltham, MA, USA) at a 1:100 dilution in blocking buffer. After several rinses in PBS, sections were incubated with fluorescein-isothiocyanate (FITC)-conjugated secondary anti-rabbit (Alexa Fluor 488, Thermo Fisher Scientific, Waltham, MA, USA) diluted 1:400 in PBS for 1 h at RT. After several washes in PBS, sections were mounted with Fluoromount (Sigma) for observation. Negative control for each assay was performed by omitting the primary antibodies or using antibodies preabsorbed with the immunizing peptide.

### 2.7. Quantitative Analysis

The images were captured using a Nikon Eclipse N*i* epifluorescence microscope and a DS-Fi3 digital camera (Nikon Instruments Ltd., Campi Bisenzio, FI, Italy). Six digital images (original magnification 20×) of both proximal and distal colon regions containing a minimum of six glands each were captured for every sample and experimental condition. PAS, HID-AB pH 2.5 stains were observed in bright light, whereas lectin binding and Muc2 immunostaining were observed in epifluorescence under 495 nm light emission. For each stain, six images per individual were considered for the quantitative analysis. In each image, six glandular crypts per type with appropriate orientation and staining were selected for counts and/or measurements. Analyses were performed by the ImageJ package (Version 2) [33] implemented with the color deconvolution plugin [23,34]. For bright-field histochemical stains (PAS, HID-AB pH 2.5), RGB images were processed by the color deconvolution procedure [35] to separate the color channels of the stain, the counterstain, and the background. Stain vectors were created from single-stain (i.e., without counterstaining) slides [34]. In the stain channel, the mean intensity was estimated for each selected cell and converted to Optical Density (OD) by the formula OD = log (255/mean intensity). For a compact representation of relative variation of sialomucins in respect to sulfomucins, mean OD values from the “blue” stain channel (related to the number of sialomucins) and the “brown”one (related to the number of sulfomucins) from color deconvolution were combined into a “sialo/sulfomucin ratio”, SSR = OD_blue_/OD_brown_ [5]. For fluorescence stains (lectins, Muc2), the Corrected Total Cell Fluorescence (CTCF) for each cell was computed [17]. Details for computing were given elsewhere [36].

### 2.8. Statistical Analysis

Statistical analyses were conducted using R software (Version 4.5.1), applying methods appropriate to the distributional and variance properties of the data. Data are presented as bar plots displaying the mean and standard deviation for each group. For inferential analyses, the Shapiro–Wilk test was used to assess normality, while both Levene’s test and Bartlett’s test were employed to evaluate homogeneity of variances. For data satisfying the assumptions of normality and equal variances, one-way analysis of variance (ANOVA) was performed, followed by Tukey’s post hoc test for multiple comparisons, or Dunnett’s test when comparing experimental groups to the CTRL. In the presence of unequal variances, Welch’s ANOVA was used, followed by the Games–Howell test for pairwise comparisons. When both normality and homogeneity of variances were violated, the non-parametric Kruskal–Wallis test was applied, followed by Dunn’s post hoc test for multiple comparisons or the Mann–Whitney U test for direct comparisons with the CTRL group. Statistical significance was defined as *p* < 0.05 and is indicated by an asterisk (*).

## 3. Results

Three mice died in the PI358934 group during the final weeks. Therefore, only nine mice were analyzed in the cited group. The final body weight was significantly higher in the HFD group (HFD: 40.1 ± 3.00; HFD + MG_13: 43.69 ± 6.08; HFD + PI: 49.91 ± 4.04) than in the control group (31.04 ± 1.04). Notably, the increase measured in the HFD + PI group was significantly higher than that in the HFD group.

### 3.1. Conventional Histochemistry

#### 3.1.1. PAS, HID-Alcian Blue pH 2.5 to Characterize Acidic and Neutral Glycoconjugates

**Figure 1:**
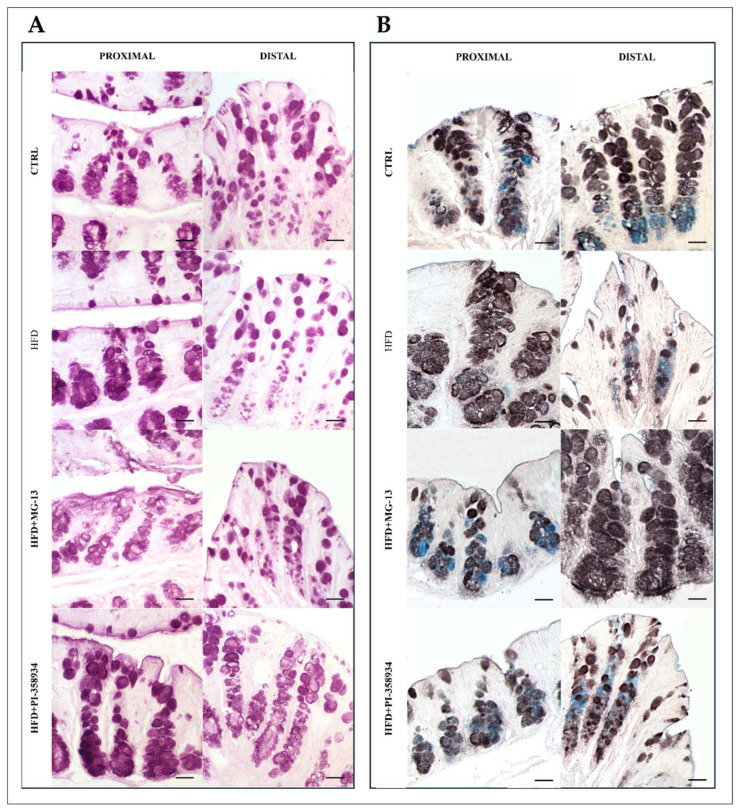
(**A**) PAS method to detect the presence of polysaccharides, glycoproteins, and other substances containing glycolic or aminohydroxyl groups in tissue samples. (**B**) High iron diamine—Alcian blue at pH 2.5 (HID–AB 2.5) to identify sulfated and nonsulfated acid glycans and to indicate the presence of sialylated and sulfated acid glycoproteins. Scale bars 20 µm.

**Figure 2:**
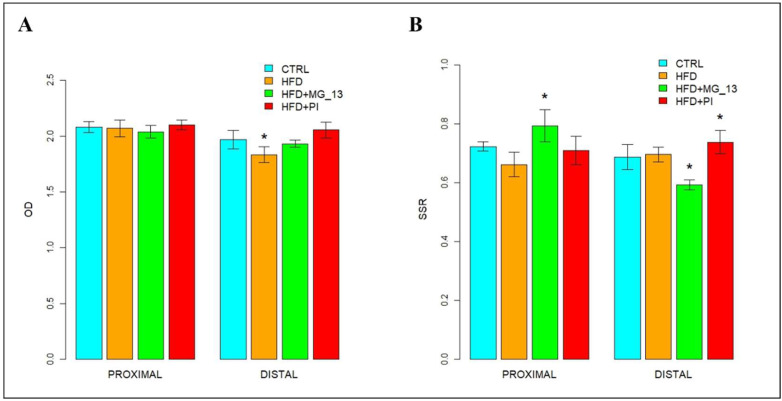
Data were shown as means ± SD of six independent experiments and analyzed by one-way ANOVA followed by Dunnett’s multiple comparisons test for PAS (**A**) and HID-AB (**B**). Asterisk indicates a statistically significant difference in mean value compared to the CTRL group (*p* < 0.05).

The glandular crypts of both the proximal and distal colon were PAS-positive through all four experimental conditions: CTRL, HFD, HFD + MG_13, and HFD + PI, indicating the presence of glycoproteins (stained in red) (Figure 1A). In the proximal colon, no statistically significant differences were observed among the groups; however, a slight increase of approximately 1% to 3% in PAS positivity was noted in the HFD + PI group compared to the other conditions. In the distal colon, a statistically significant reduction in PAS positivity was observed in the HFD group compared to both the CTRL (7%) and HFD + PI (11%) groups (Figure 2A).

Goblet cells in the glandular crypts of the proximal and distal colon in all four experimental conditions were stained with Alcian blue pH 2.5 (blue) and HID (brown), indicating the presence of sialylated and sulfated acid glycoproteins. In both the proximal and distal colon, goblet cells were stained more intensely with HID in the upper part of the crypts, whereas Alcian blue pH 2.5 showed stronger staining in the lower part of the crypts (Figure 1B). In the proximal colon, a statistically significant increase of approximately 9%, 16%, and 10% in the SSR was, respectively, observed in the HFD + MG_13 group compared to CTRL, HFD, and HFD + PI. In the distal colon, a statistically significant decrease in the SSR was observed in HFD + MG_13 compared to CTRL (14%), HFD (16%), and HFD + PI (20%).

Furthermore, a statistically significant increase in SSR was observed only in HFD + PI compared to HFD + MG_13 (Figure 2B). In HFD + PI mice, a strong reduction in HID positivity is observed along the glandular crypts of the proximal colon, indicating a significant decrease in mucin sulfation. Conversely, in the distal colon under the same condition, goblet cells were mainly HID-positive in the upper part of the crypts, with Alcian blue staining at pH 2.5 being more evident in the lower part of the crypts (Figure 2B).

#### 3.1.2. Lectin Histochemistry

##### UEA-I for the Identification of α (1,2)-Linked Fucose in O-Linked Glycans

**Figure 3:**
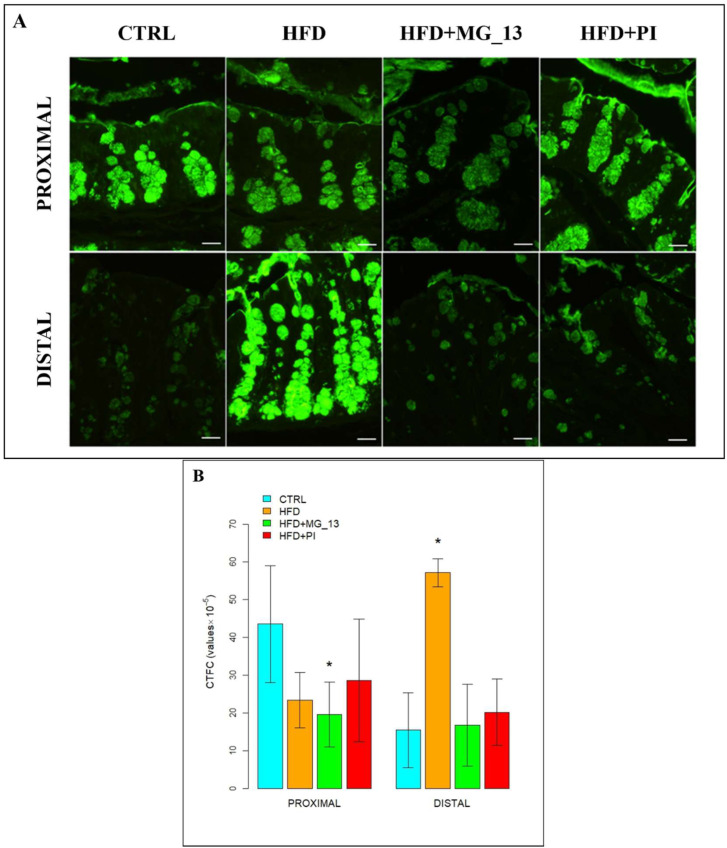
(**A**) Staining with UEA-I FITC lectin to detect α (1,2)-linked fucose in O-linked glycans. Scale bars 20 µm. (**B**) Data were shown as means ± SD of six independent experiments and analyzed by one-way ANOVA followed by Dunnett’s multiple comparisons test for UEA-I FITC. Asterisk indicates a statistically significant difference in mean value compared to the CTRL group (*p* < 0.05).

In the proximal and distal colon of CTRL mice, goblet cells were intensely bound with UEA-I throughout the glandular crypts, but the lectin positivity decreased in distal CTRL (Figure 3A). In the proximal colon of HFD mice, a reduction of 46% in UEA-I staining was observed in goblet cells compared to CTRL. A statistically significant decrease of approximately 55% in positivity was also observed in the proximal colon of HFD + MG_13 mice compared to CTRL. In the distal colon, muciparous goblet cells throughout the glandular crypt of HFD mice showed an increased positivity of 73% compared to the CTRL (Figure 3B).

##### AAL for the Identification of α (1,6)-Linked Fucose in O-Linked Glycans

**Figure 4:**
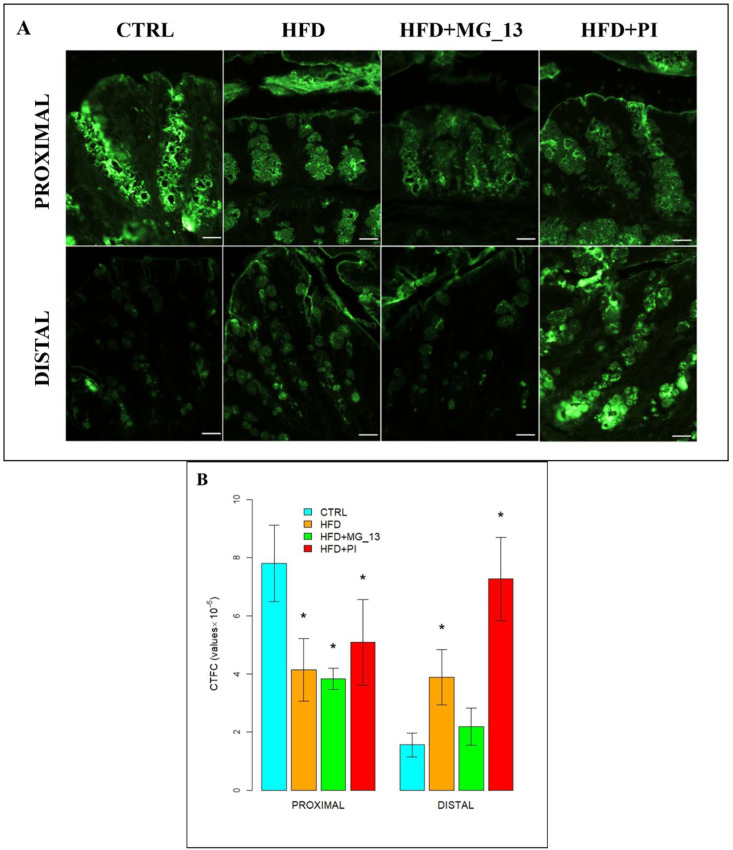
(**A**) Staining with AAL-FITC lectin to detect α(1,6)-linked fucose in O-linked glycans. Scale bars 20 µm. (**B**) Data were shown as means ± SD of six independent experiments and analyzed by one-way ANOVA for distal measurements (Kruskal–Wallis test for proximal ones), followed by Dunnett’s (Mann–Whitney’s U) test for multiple comparisons for AAL-FITC. Asterisk indicates a statistically significant difference in mean value compared to the CTRL group (*p* < 0.05).

In the proximal and distal colon of CTRL mice, goblet cells were bound with AAL throughout the glandular crypts (Figure 4A). In the proximal colon, a statistically significant decrease of 47%, 51%, and 35% was observed, respectively, in HFD, HFD + MG-13, and HFD + PI compared to CTRL. In the distal colon, a statistically significant increase was observed in HFD (60%) and HFD + PI (79%) compared to CTRL (Figure 4B).

##### WGA for the Identification of *N*-Acetylglucosamine in O-Linked Glycans

**Figure 5:**
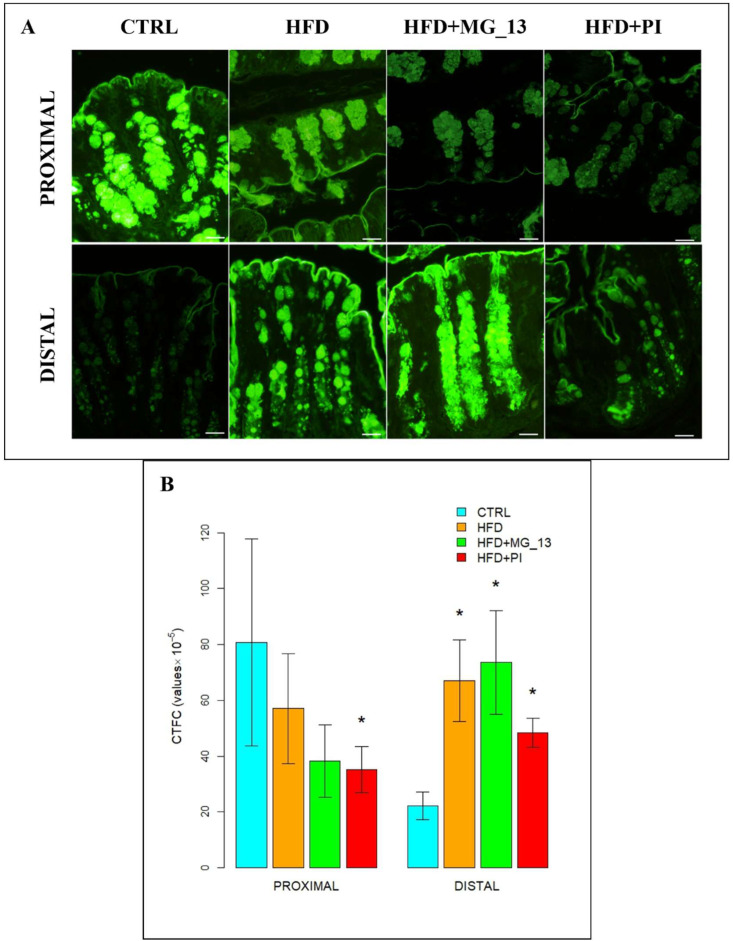
(**A**) Staining with WGA-FITC lectin to detect *N*-acetylglucosamine in O-linked glycans. Scale bars 20 µm. (**B**) Data were shown as means ± SD of six independent experiments and analyzed by the Kruskal–Wallis test followed by Mann–Whitney’s U multiple comparisons test for WGA-FITC. Asterisk indicates a statistically significant difference in mean value compared to the CTRL group (*p* < 0.05).

In the proximal and distal colon of CTRL mice, goblet cells were stained intensely with WGA throughout the glandular crypts, but lectin positivity decreased in distal CTRL (Figure 5A). In the proximal colon, a statistically significant decrease of 56% in positivity was observed in HFD + PI compared to CTRL. WGA positivity decreases in HFD and HFD + MG-13 compared to CTRL. In the distal colon, muciparous goblet cells throughout the glandular crypt of HFD, HFD + MG-13, and HFD + PI mice showed statistically significant increased positivity from 54% to 70% compared to the CTRL (Figure 5B).

##### SBA for the Identification of *N*-Acetylgalactosamine in O-Linked Glycans

**Figure 6:**
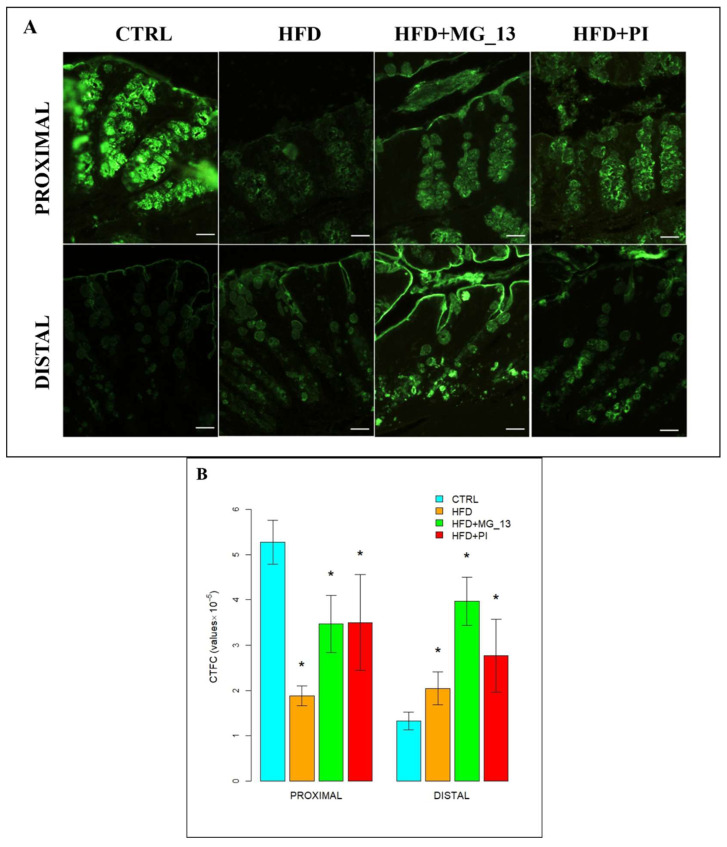

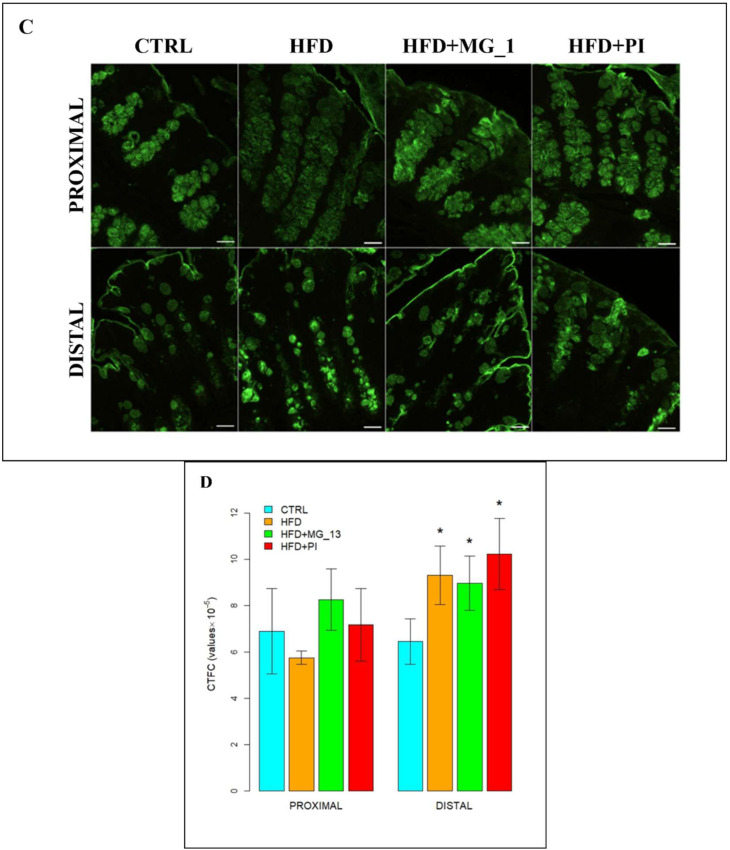
(**A**) Staining with SBA-FITC lectin to detect *N*-acetylgalactosamine in O-linked glycans. Scale bars 20 µm. (**B**) Data were shown as means ± SD of six independent experiments and analyzed by one-way ANOVA followed by Dunnett’s multiple comparisons test for SBA-FITC. Asterisk indicates a statistically significant difference in mean value compared to the CTRL group (*p* < 0.05). (**C**) Enzymatic digestion with sialidase and staining with the lectin SBA-FITC to detect terminal sialic acid linked to *N*-acetylgalactosamine in O-linked glycans. Scale bars 20 µm. (**D**) Data are shown as means ± SD of six independent experiments and analyzed by one-way ANOVA followed by Dunnett’s multiple comparisons test for sialidase/SBA-FITC. Asterisk indicates a statistically significant difference in mean value compared to the CTRL group (*p* < 0.05).

In the proximal and distal colon of CTRL mice, goblet cells were stained intensely with SBA throughout the glandular crypts, but lectin positivity decreased in distal CTRL (Figure 6A). A statistically significant decrease in SBA positivity was observed in the proximal colon of HFD mice (64%) compared to CTRL and mice fed with HFD + PI (34%). Also, a statistically significant decrease of 34% was observed in HFD + MG_13 compared to CTRL. In the distal colon, SBA was statistically significantly increased from 30% to 67% in HFD + MG_13 compared to the other conditions, and in HFD (35%) and HFD + PI (52%) compared to CTRL (Figure 6B). To indirectly demonstrate the presence of sialic acid, we used digestion with sialidase/SBA. A significant increase in positivity from 28% to 37%, indicating the presence of terminal sialic acid bound to *N*-acetylgalactosamine, was observed in experimental conditions compared to the CTRL in the distal colon. In the proximal colon, an increase was observed in HFD + MG_13 (17%) and HFD + PI (4%), although it was not statistically significant.

##### PNA for the Identification of Terminal Galactose β1,3*N*-Acetylgalactosamine in O-Linked Glycans

**Figure 7:**
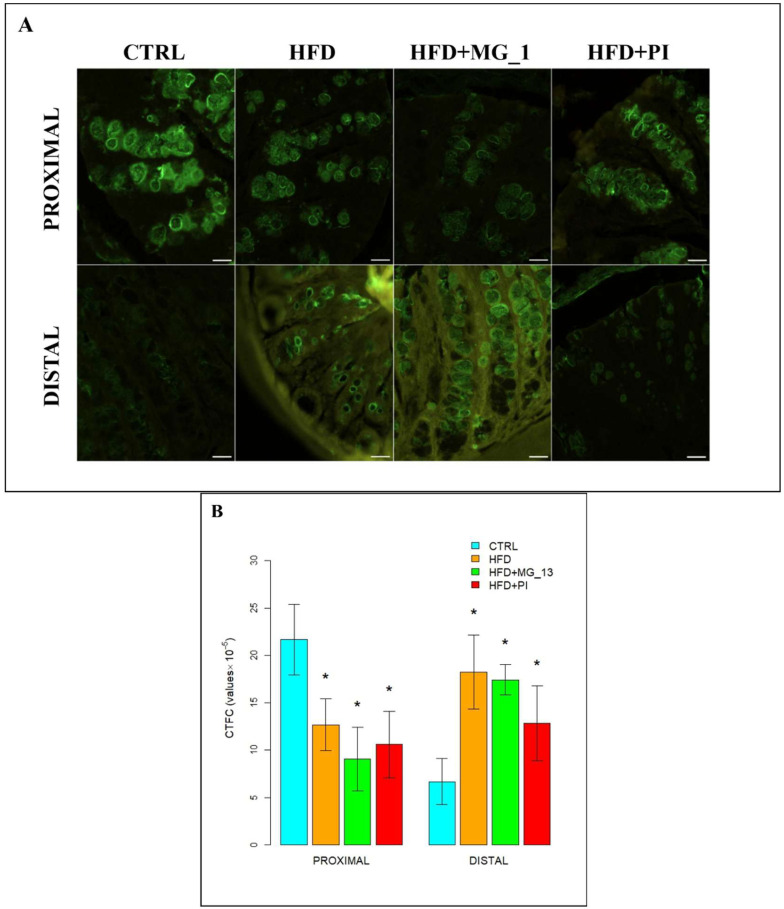
(**A**) Desulfation treatment and staining with the PNA-FITC lectin to detect terminal Galactose in O-linked glycans. Scale bars 20 µm. (**B**) Data are shown as means ± SD of six independent experiments and analyzed by one-way ANOVA followed by Dunnett’s multiple comparisons test for the desulfation treatment/PNA-FITC. Asterisk indicates a statistically significant difference in mean value compared to the CTRL group (*p* < 0.05).

In the proximal and distal colon, the PNA binding was not evident in the whole glandular crypts, in the four different experimental conditions. So, it is followed by the application of the desulfation treatment/PNA, a method which eliminates the masking of sulphate groups that can create steric clutter to the lectin bond. After desulfation, an increase in lectin positivity is observed in CTRL of the proximal colon (Figure 7A). Under the experimental conditions, a significant reduction from 42% to 58% was observed in HFD, HFD + PI, and particularly in HFD + MG_13 (Figure 7B) compared to CTRL. In the distal colon, a significant increase from 48% to 63% in lectin positivity was observed in HFD, HFD + MG_13, and HFD + PI compared to the CTRL (Figure 7B).

##### ConA for the Identification of D-Mannose in O-Linked Glycans

**Figure 8:**
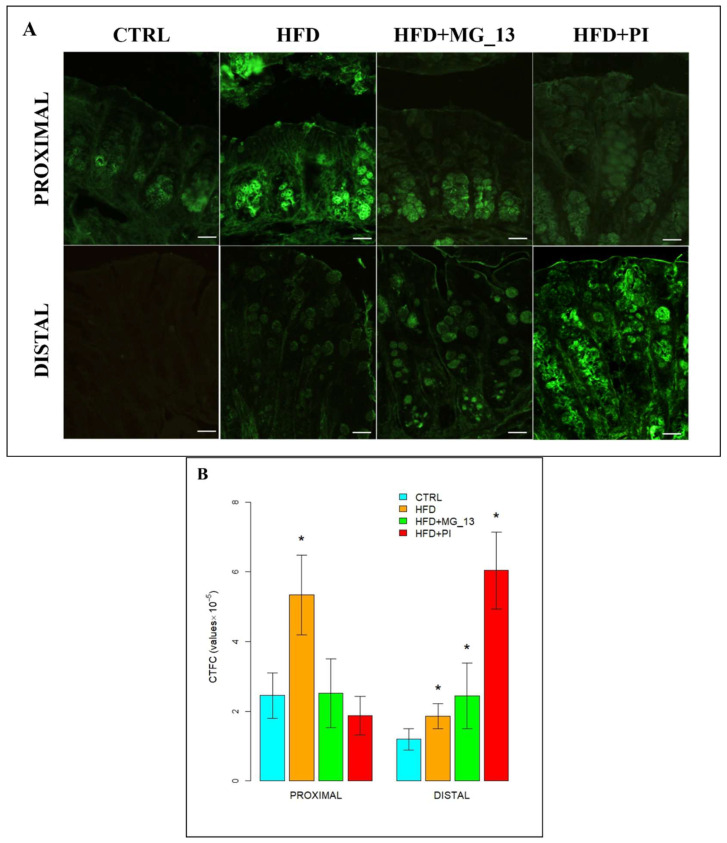
(**A**) Staining with ConA-FITC lectin to detect D-Mannose in O-linked glycans. Scale bars 20 µm. (**B**) Data were shown as means ± SD of six independent experiments and analyzed by one-way ANOVA followed by Dunnett’s multiple comparisons test for ConA-FITC. Asterisk indicates a statistically significant difference in mean value compared to the CTRL group (*p* < 0.05).

In the proximal and distal colon of CTRL mice, goblet cells were stained intensely with ConA throughout the glandular crypts, but the lectin positivity decreases in distal CTRL (Figure 8A). In the proximal colon, a statistically significant increase of 54% is observed in ConA positivity in HFD compared to CTRL. In the distal colon ConA, a statistically significant increase from 36% to 80% was observed in HFD mice and in the two chickpea accessions compared to CTRL (Figure 8B).

### 3.2. Immunohistochemistry Anti-Muc2

**Figure 9:**
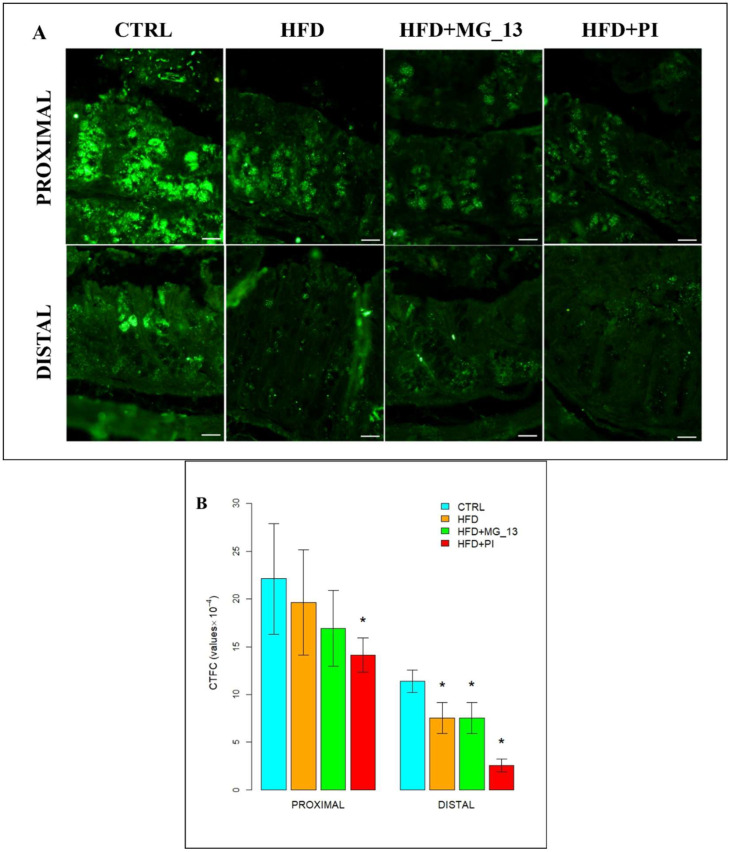
(**A**) Muc2-positive goblet cells in proximal and distal colon. Scale bars 20 µm. (**B**) Data were shown as means ± SD of six independent experiments and analyzed by one-way ANOVA followed by Dunnett’s test for multiple comparisons. Asterisk indicates a statistically significant difference in mean value compared to the CTRL group (*p* < 0.05).

The immunohistochemical study was carried out to evaluate the expression of Muc2 in the proximal and distal colon (Figure 9A). In the proximal colon of CTRL mice, Muc2 was expressed along the entire glandular crypt. In HFD, HFD + MG-13, and HFD + PI mice, the expression of Muc2 is weakly diffused. In HFD and HFD + MG-13 mice, a weak reduction of Muc2 expression, respectively, of 11% and 23%, was observed compared to CTRL, while in HFD + PI mice, the expression was significantly reduced by 36% (Figure 9B). In the distal colon of CTRL mice, Muc2 was less expressed compared to the CTRL proximal colon. In HFD and HFD + MG-13 mice a reduction of Muc2 expression of 34% was observed to compare to CTRL, and in HFD + PI mice the expression was greatly reduced (78%) (Figure 9B).

## 4. Discussion

Our findings indicate that a HFD markedly reduces Muc2 expression in both the proximal and distal colon, likely through the induction of a pro-inflammatory state [6,37,38,39]. Supplementation with chickpea accessions, particularly MG_13, partially counteracted these effects, improving Muc2 levels and normalizing blood parameters (glucose, triglycerides, AST, NF-κB) toward control values [15]. This protective activity may be related to the antioxidant and anti-inflammatory properties of legumes, including polyphenol- and carotenoid-mediated modulation of oxidative stress and glycosylation pathways, which support goblet cell maturation and mucin production, thereby preserving mucosal barrier integrity [40,41]. 

Using conventional histochemical methods, we demonstrated the presence of both sialo- and sulfomucins in the proximal and distal colon under all conditions. Interestingly, we observed an increased sialo/sulfomucin ratio (SSR) in the proximal colon of HFD + MG_13 mice. Considering the role of the proximal colon in water absorption, fermentation, and immune regulation [42], this suggests that MG_13 may promote a more hydrated and selectively favorable environment for beneficial bacteria. Increased mucin sialylation may specifically modulate immune cell activity or improve barrier function against the destructive effects of a HFD [43]. The reduction in SSR with MG_13 could reflect a shift in microbial metabolism toward sulfated mucins or consumption of sialylated mucins. Conversely, the higher SSR in the HFD + PI group may indicate a distinct microbial profile or an unresolved inflammatory state, possibly linked to the higher linoleic acid content of this diet [15]. While linoleic acid could promote inflammation, the concurrent increase in sialylation may represent a compensatory mechanism to protect the mucosa from oxidative and inflammatory damage [43,44]. These two phenomena might constitute complementary aspects of an adaptive response. 

Lectin histochemistry revealed differences in glycan binding patterns in the mucus of goblet cells across the four dietary groups. Fucose expression detected by AAL decreased in the HFD, HFD + MG_13, and HFD + PI groups in both colonic segments, while UEA-I staining decreased in the proximal colon of HFD + MG_13 mice and increased in the HFD + PI group (though not significantly). In the distal colon, UEA-I positivity increased in the HFD group compared to CTRL. Fucosylated glycans are crucial for maintaining mucus viscosity, supporting native microbial communities, and protecting the mucosal barrier [45]. Their reduction in the HFD + MG_13 group could reflect polyphenol-mediated attenuation of inflammation, consistent with Saldova et al. (2024) [46], who reported altered fucosylation in mice with compromised barrier function. This suggests that reduced UEA-I staining in our model might indicate changes in the mucus layer composition and gut microbiota, contributing to the observed anti-inflammatory effects of the HFD + MG_13 diet. 

Chickpeas are rich in proteins, fibers, and bioactive compounds such as polyphenols, which lower lipid levels, regulate blood pressure, reduce insulin resistance, and alleviate systemic inflammation [40]. In the MG_13 diet, increased colonic *N*-acetylglucosamine (GlcNAc), together with polyphenols, may modulate the gut microbiota and reinforce the intestinal barrier [4,41,47]. GlcNAc is a key component of intestinal mucus that protects the mucosa from direct bacterial contact [4]. Although an uncontrolled rise in GlcNAc under HFD may promote inflammation and vascular dysfunction, its increase in the context of a balanced or polyphenol-enriched diet appears beneficial, strengthening the mucus layer, reducing inflammation, and lowering chronic disease risk. This context-dependent duality highlights GlcNAc as a potential biomarker and therapeutic target for counteracting diet-induced metabolic and vascular alterations. 

GalNAc residues significantly decreased in the proximal colon of HFD-fed mice consistent with studies reporting increased GalNAc in Crohn’s disease (CD) [48], while they increased in the distal colon under HFD, likely reflecting diet-induced inflammatory responses and metabolic dysfunctions in glycan synthesis [49]. We also detected terminal sialic acid (via sialidase/SBA treatment), which increased in mice [5,43] fed chickpea-enriched diets. Sialic acid contributes to mucosal integrity and immune modulation; its loss under HFD may compromise the barrier and favor inflammation, while its increase with chickpeas likely reflects beneficial stimulation of secretion and protection from diet-induced damage [5,43]. 

Mannose (ConA) accumulated mainly in the distal colon, possibly due to lower absorption in the small intestine. This sugar may benefit gut health by modulating inflammation and supporting epithelial healing through normalization of protein *N*-glycosylation and prevention of TNF-α-induced ER stress [50,51]. 

Finally, HFD-induced oxidative stress, driven by increased ROS production, can disrupt mucin glycosylation and goblet cell maturation [52]. The antioxidant compounds in legumes, including polyphenols and carotenoids, may reduce ROS, restore glycosylation balance, and support effective mucin production [40,53]. MG_13 supplementation may further enhance this effect by promoting glycosyltransferase expression and maintaining mucoproteins in a functional state, counteracting the detrimental effects of HFD on mucosal barrier integrity.

## 5. Conclusions

In conclusion, this study demonstrates that HFD significantly decreases Muc2 expression and alters mucin composition in the colon. The addition of chickpea accessions, especially MG_13, appears to exert a compensatory effect, evidenced by improvements Muc2 expression, and the oligosaccharide profile of mucins suggesting potential modulatory effects on the mucosal barrier. These findings support the hypothesis that dietary interventions, specifically using select chickpea accessions like MG_13, can mitigate the adverse effects of a HFD on mucin glycosylation. However, it is important to note that these results were observed in an animal model, and further mechanistic validation and phytochemical quantification are needed to fully understand the implications of these changes.

## Figures and Tables

**Table 1:** Lectins employed with their diluting buffers, working dilutions, and inhibiting sugars. Binding specificities: PNA: Galβ1,3GalNAc; SBA: GalNAc; WGA: (GlcNAcβ1,4)n; UEA I: Fucα1,2; AAL: Fucα1,6GlcNAcβNAsn; Fucα1,3,Fucα1,4; ConA: D-Man, D-Glc [25,26,27,28,29,30].

Lectin	Origin	Buffer	Dilution	Inhibitory Sugar Conc.
PNA	*Arachis hypogaea*	Hepes	10 mg/mL	0.2 M Gal
SBA	*Glycine max*	Hepes	20 mg/mL	0.2 M GalNAc
WGA	*Triticum vulgare*	Hepes	20 mg/mL	0.5 M GlcNAc
UEA-I	*Ulex europaeus*	Hepes	10 mg/mL	0.2 M L-Fuc
AAL	*Aleuria aurantia*	Hepes	10 mg/mL	0.2 M L-Fuc
ConA	*Canavalia ensiformis*	Hepes	20 mg/mL	0.1 M MαM

## Data Availability

The results of the statistical analysis are available in the Supplementary Materials.

## Supplementary Materials

The following supporting information can be downloaded at https://www.mdpi.com/article/10.3390/nu17193035/s1, Table S1: Statistical comparisons of proximal OD SSR values between controls (CTRL), high-fat diet (HFD), HFD + MG_13, and HFD + PI groups. Table S2: Statistical comparisons of distal OD SSR values between controls (CTRL), high-fat diet (HFD), HFD + MG_13, and HFD + PI groups. Table S3: Statistical comparisons of proximal and distal CTFC × 10^−5^ values between controls (CTRL), high-fat diet (HFD), HFD + MG_13, and HFD + PI groups. Table S4: Statistical comparisons of proximal OD values between controls (CTRL), high-fat diet (HFD), HFD + MG_13, and HFD + PI groups. Table S5: Statistical comparisons of distal OD values between controls (CTRL), high-fat diet (HFD), HFD + MG_13, and HFD + PI groups. Table S6: Statistical comparisons of proximal CTFC × 10^−4^ values between controls (CTRL), high-fat diet (HFD), HFD + MG_13, and HFD + PI groups. Table S7: Statistical comparisons of distal CTFC × 10^−4^ values between controls (CTRL), high-fat diet (HFD), HFD + MG_13, and HFD + PI groups.
