# Supplementary material for: Mucin Alterations in Response to High-Fat Diet and the Potential Protective Role of Chickpea Accessions
Source: Nutrients. 2025 Sep 23;17(19):3035. doi: 10.3390/nu17193035 (PMC12526196; doi:10.3390/nu17193035)
Supplement: Supplementary file 1 [file nutrients-17-03035-s001.zip › nutrients-3837509-supplementary.pdf]

OD SSR AB2.5/HID

Table S1. *Statistical comparisons of* ***proximal*** *OD SSR values between controls (CTRL), high-fat diet (HFD), HFD+MG_13, and HFD+PI groups.*

OD SSR AB2.5/HID	Mean ± SD	F (p-value)	SW (p-value)	B (p-value)	T (p-values)	D p-value
CTRL	0.72 ± 0.02	9.624 (.0004*)	0.968 (.621)	6.114 (.106)	HFD (.097), HFD+MG_13 (.045*), HFD+PI (.953)	-
HFD	0.66 ± 0.04				CTRL (.097), HFD+MG_13 (.0001*), HFD+PI (.244)	.059
HFD+MG_13	0.79 ± 0.05				CTRL (.045*), HFD (.0001*), HFD+PI (.015*)	.026*
HFD+PI	0.71 ± 0.05				CTRL (.953), HFD (.244), HFD+MG_13 (.015*)	.915

Table S2. *Statistical comparisons of* ***distal*** *OD SSR values between controls (CTRL), high-fat diet (HFD), HFD+MG_13, and HFD+PI groups.*

OD SSR AB2.5/HID	Mean ± SD	F (p-value)	SW (p-value)	B (p-value)	T (p-values)	D p-value
CTRL	0.69 ± 0.04	21.42 (1.86e-06*)	0.945 (.206)	4.689 (.196)	HFD (.975), HFD+MG_13 (.0003*), HFD+PI (.068)	-
HFD	0.70 ± 0.02				CTRL (.975), HFD+MG_13 (.0001*), HFD+PI (.150)	.953
HFD+MG_13	0.59 ± 0.02				CTRL (.0003*), HFD (.0001*), HFD+PI (.000001*)	.001*
HFD+PI	0.74 ± 0.04				CTRL (.068), HFD (.150), HFD+MG_13 (.000001*)	.041*

Abbreviations: OD, optical density; AB, alcian blue; HID, high iron diamine; SD, standard deviation; SW, Shapiro-Wilk test for normality of distribution; B, Bartlett's test for homogeneity of variances; F, ANOVA test; T, Tukey’s test for multiple comparisons of means; D, Dunnett’s test for multiple comparisons of means with the CTRL group. *The test yields a statistically significant p-value ($p < 0.05$).

LECTINS

Table S3. *Statistical comparisons of* ***proximal*** *and* ***distal*** *CTFC* $\times 10^{-5}$ *values between controls (CTRL), high-fat diet (HFD), HFD+MG_13, and HFD+PI groups.*

LECTIN			Mean ± SD	F (p-value)	KW (p-value)	SW (p-value)	B (p-value)	T or DD (p-values)	D or U p-value
**UEA-I**									
	Proximal	CTRL	43.46 ± 15.5	4.282^{L} (.017*)	-	0.943^{L} (.187)	1.902^{L} (.593)	HFD (.085), HFD+MG_13 (.012*), HFD+PI (.204)	-
		HFD	23.33 ± 7.35					CTRL (.085), HFD+MG_13 (.789), HFD+PI (.962)	.051
		HFD+MG_13	19.54 ± 8.64					CTRL (.012*), HFD (.789), HFD+PI (.506)	.006*
		HFD+PI	28.53 ± 16.22					CTRL (.204), HFD (.962), HFD+MG_13 (.506)	.131
	Distal	CTRL	15.42 ± 9.93	30.89 (1e-07*)	-	0.935 (.125)	4.672 (.197)	HFD (4e-07*), HFD+MG_13 (.994), HFD+PI (.784)	-
		HFD	57.07 ± 3.77					CTRL (4e-07*), HFD+MG_13 (7e-07*), HFD+PI (3e-06*)	.001*
		HFD+MG_13	16.68 ± 10.85					CTRL (.994), HFD (7e-07*), HFD+PI (.900)	.989
		HFD+PI	20.18 ± 8.82					CTRL (.784), HFD (3e-06*), HFD+MG_13 (.900)	.674
**WGA**									
	Proximal	CTRL	80.82 ± 37.1	-	10.813 (.013*)	0.966 (.570)	10.704 (.013*)	HFD (1.00), HFD+MG_13 (.203), HFD+PI (.020*)	-
		HFD	57.04 ± 19.79					CTRL (1.00), HFD+MG_13 (.850), HFD+PI (.133)	.394
		HFD+MG_13	38.25 ± 13					CTRL (.203), HFD (.850), HFD+PI (1.00)	.065
		HFD+PI	35.18 ± 8.25					CTRL (.020*), HFD (.133), HFD+MG_13 (1.00)	.004*
	Distal	CTRL	22.1 ± 4.95	-	18.847 (.0003*)	0.882 (.008*)	11.004 (.012*)	HFD (.003*), HFD+MG_13 (.0009*), HFD+PI (.725)	-
		HFD	67.04 ± 14.7					CTRL (.003*), HFD+MG_13 (1.00), HFD+PI (.330)	.002*
		HFD+MG_13	73.57 ± 18.54					CTRL (.0009*), HFD (1.00), HFD+PI (.148)	.002*
		HFD+PI	48.35 ± 5.26					CTRL (.725), HFD (.330), HFD+MG_13 (.148)	.002*
**AAA**									
	Proximal	CTRL	7.8 ± 1.32	-	14.70 (.002*)	0.902 (.024*)	7.334 (.062)	HFD (.007*), HFD+MG_13 (.005*), HFD+PI (.475)	-
		HFD	4.15 ± 1.09					CTRL (.007*), HFD+MG_13 (1.00), HFD+PI (.850)	.002*
		HFD+MG_13	3.84 ± 0.36					CTRL (.005*), HFD (1.00), HFD+PI (.668)	.002*
		HFD+PI	5.09 ± 1.47					CTRL (.475), HFD (.850), HFD+MG_13 (.668)	.015*
	Distal	CTRL	1.56 ± 0.42	44.14 (5e-09*)	-	0.962 (.476)	7.158 (.067)	HFD (.002*), HFD+MG_13 (.658), HFD+PI (<.0001*)	-
		HFD	3.9 ± 0.95					CTRL (.002*), HFD+MG_13 (.025*), HFD+PI (<.0001*)	.0009*
		HFD+MG_13	2.19 ± 0.64					CTRL (.658), HFD (.025*), HFD+PI (<.0001*)	.527
		HFD+PI	7.27 ± 1.44					CTRL (<.0001*), HFD (<.0001*), HFD+MG_13 (<.0001*)	.0001*

**SBA**								
Proximal	CTRL	5.28 ± 0.49					HFD (5e-06*), HFD+MG_13 (.001*), HFD+PI (.029*)	-
	HFD	1.88 ± 0.22	76.496^{W} (4e-07*)	-	0.966 (.562)	9.644 (.022*)	CTRL (5e-06*), HFD+MG_13 (.004*), HFD+PI (.045*)	.002*
	HFD+MG_13	3.47 ± 0.63					CTRL (.001*), HFD (.004*), HFD+PI (1.00)	.002*
	HFD+PI	3.5 ± 1.06					CTRL (.029*), HFD (.045*), HFD+MG_13 (1.00)	.015*
Distal	CTRL	1.33 ± 0.2					HFD (.012*), HFD+MG_13 (7.31e-05*), HFD+PI (.025*)	-
	HFD	2.05 ± 0.36	42.822^{W} (5e-06*)	-	0.964 (.538)	8.374 (.039*)	CTRL (.012*), HFD+MG_13 (.0002*), HFD+PI (.279)	.009*
	HFD+MG_13	3.97 ± 0.53					CTRL (7.31e-05*), HFD (.0002*), HFD+PI (.059)	.002*
	HFD+PI	2.77 ± 0.81					CTRL (.025*), HFD (.279), HFD+MG_13 (.059)	.004*
**PNA**								
Proximal	CTRL	0.77 ± 0.77					HFD (1.00), HFD+MG_13 (.002*), HFD+PI (.247)	-
	HFD	0.8 ± 0.25	-	16.227 (.001*)	0.809 (.0004*)	10.645 (.014*)	CTRL (1.00), HFD+MG_13 (.008*), HFD+PI (.615)	.178
	HFD+MG_13	2.94 ± 0.81					CTRL (.002*), HFD (.008*), HFD+PI (.725)	.008*
	HFD+PI	2 ± 0.23					CTRL (.247), HFD (.615), HFD+MG_13 (.725)	.065
Distal	CTRL	0.81 ± 0.34					HFD (.043*), HFD+MG_13 (.997), HFD+PI (.046*)	-
	HFD	1.41 ± 0.32	9.982^{W} (.003*)	-	0.976 (.811)	17.594 (.0005*)	CTRL (.043*), HFD+MG_13 (.014*), HFD+PI (.297)	.015*
	HFD+MG_13	0.78 ± 0.1					CTRL (.997), HFD (.014*), HFD+PI (.045*)	.699
	HFD+PI	2.16 ± 0.89					CTRL (.046*), HFD (.297), HFD+MG_13 (.045*)	.009*
**ConA**								
Proximal	CTRL	2.45 ± 0.65					HFD (.003*), HFD+MG_13 (.999), HFD+PI (.384)	-
	HFD	5.34 ± 1.15	13.243^{W} (.0006*)	-	0.979 (.8742)	3.150 (.369)	CTRL (.003*), HFD+MG_13 (.005*), HFD+PI (.001*)	.002*
	HFD+MG_13	2.52 ± 0.99					CTRL (.999), HFD (.005*), HFD+PI (.538)	.937
	HFD+PI	1.87 ± 0.55					CTRL (.384), HFD (.001*), HFD+MG_13 (.538)	.180
Distal	CTRL	1.19 ± 0.31					HFD (.004*), HFD+MG_13 (.250), HFD+PI (.0004*)	-
	HFD	1.85 ± 0.36	31.767^{W} (1e-05*)	-	0.962 (.476)	7.158 (.067)	CTRL (.004*), HFD+MG_13 (.024*), HFD+PI (.005*)	.002*
	HFD+MG_13	2.44 ± 0.94					CTRL (.250), HFD (.024*), HFD+PI (.0004*)	.065*
	HFD+PI	6.04 ± 1.10					CTRL (.0004*), HFD (.005*), HFD+MG_13 (.0004*)	.002*
**SIALIDASI-PNA**								
Proximal	CTRL	2.83 ± 2.34					HFD (.998), HFD+MG_13 (.002*), HFD+PI (.950)	-
	HFD	2.68 ± 1.04	8.829 (.0006*)	-	0.954 (.341)	2.938 (.401)	CTRL (.998), HFD+MG_13 (.001*), HFD+PI (.902)	.998
	HFD+MG_13	7.08 ± 1.70					CTRL (.002*), HFD (.001*), HFD+PI (.006*)	0.001*
	HFD+PI	3.35 ± 1.53					CTRL (.950), HFD (.902), HFD+MG_13 (.006*)	.910
Dis	CTRL	3.56 ± 1.99	-				HFD (.330), HFD+MG_13 (1.00), HFD+PI (.002*)	-
	HFD	6.35 ± 1.86		14.993	0.896	7.850	CTRL (.330), HFD+MG_13 (1.00), HFD+PI (.615)	.041*

	HFD+MG_13	4.69 ± 1.31		(.002*)	(.017*)	(.049*)	CTRL (1.00), HFD (1.00), HFD+PI (.017*)	.394
	HFD+PI	10.86 ±4.36					CTRL (.002*), HFD (.615), HFD+MG_13 (.017*)	.002*
**SIALIDASI-SBA**								
Proximal	CTRL	6.89 ± 1.84					HFD (.499), HFD+MG_13 (.482), HFD+PI (.991)	-
	HFD	5.76 ± 0.29	7.687^{W}	-	0.943	11.134	CTRL (.499), HFD+MG_13 (.018*), HFD+PI (.242)	.589
	HFD+MG_13	8.26 ± 1.31	(.008*)		(.187)	(.011*)	CTRL (.482), HFD (.018*), HFD+PI (.584)	.180
	HFD+PI	7.18 ± 1.57					CTRL (.991), HFD (.242), HFD+MG_13 (.584)	.589
Distal	CTRL	6.45 ± 0.97					HFD (.004*), HFD+MG_13 (.012*), HFD+PI (.0002*)	-
	HFD	9.31 ± 1.27	9.983	-	0.963	1.008	CTRL (.004*), HFD+MG_13 (.966), HFD+PI (.584)	.002*
	HFD+MG_13	8.97 ± 1.17	(.0003*)		(.510)	(.799)	CTRL (.012*), HFD (.966), HFD+PI (.328)	.006*
	HFD+PI	10.23 ± 1.54					CTRL (.0002*), HFD (.584), HFD+MG_13 (.328)	<.001*
**DESOLF PNA**								
Proximal	CTRL	21.67 ± 3.7					HFD (.0008*), HFD+MG_13 (.00001*), HFD+PI (.00007*)	-
	HFD	12.67 ± 2.73	17.04	-	0.957	0.453	CTRL (.0008*), HFD+MG_13 (.274), HFD+PI (.709)	.0004*
	HFD+MG_13	9.06 ± 3.39	(9e-06*)		(.377)	(.929)	CTRL (.00001*), HFD (.274), HFD+PI (.856)	.0004*
	HFD+PI	10.59 ± 3.5					CTRL (.00007*), HFD (.709), HFD+MG_13 (.856)	.0004*
Distal	CTRL	6.68 ± 2.43					HFD (<.0001*), HFD+MG_13 (<.0001*), HFD+PI (.014*)	-
	HFD	18.26 ± 3.91	17.12	-	0.979	4.450	CTRL (<.0001*), HFD+MG_13 (.968), HFD+PI (.034*)	<.0001*
	HFD+MG_13	17.43 ± 1.60	(9e-06*)		(.878)	(.217)	CTRL (<.0001*), HFD (.968), HFD+PI (.085)	<.0001*
	HFD+PI	12.83 ± 3.97					CTRL (.014*), HFD (.034*), HFD+MG_13 (.085)	.008*

Abbreviations: CTFC, corrected total cell fluorescence; SD, standard deviation; L, test performed on log-transformed data; SW, Shapiro-Wilk test for normality of distribution; B, Bartlett's test for homogeneity of variances; F, ANOVA test; T, Tukey's test after ANOVA for multiple comparisons of means; D, Dunnett's test for multiple comparisons of means with the CTRL group; W, Welch ANOVA followed by Games-Howell test for multiple comparisons of means; KW, Kruskal-Wallis rank sum test; DD, Dunn's test after KW for multiple comparisons; U, Mann–Whitney's U test versus CTRL group. *The test yields a statistically significant p-value ($p < 0.05$).

PAS

Table S4. *Statistical comparisons of **proximal** OD values between controls (CTRL), high-fat diet (HFD), HFD+MG_13, and HFD+PI groups.*

**PAS**	Mean ± SD	F (p-value)	SW (p-value)	B (p-value)	T (p-values)	D p-value
CTRL	2.08 ± 0.05	1.197 (.336)	0.958 (.398)	1.642 (.650)	-	-
HFD	2.07 ± 0.08				-	.986
HFD+MG_13	2.04 ± 0.06				-	.478
HFD+PI	2.10 ± 0.04				-	.868

Table S5. *Statistical comparisons of **distal** OD values between controls (CTRL), high-fat diet (HFD), HFD+MG_13, and HFD+PI groups.*

**PAS**	Mean ± SD	F (p-value)	SW (p-value)	B (p-value)	T (p-values)	D p-value
CTRL	1.97 ± 0.08	10.98 (.0001*)	0.984 (.956)	3.794 (.285)	HFD (.011*), HFD+MG_13 (.750), HFD+PI (.172)	-
HFD	1.83 ± 0.07				CTRL (.011*), HFD+MG_13 (.092), HFD+PI (.00009*)	.006*
HFD+MG_13	1.93 ± 0.03				CTRL (.750), HFD (.092), HFD+PI (.024*)	.631
HFD+PI	2.05 ± 0.07				CTRL (.172), HFD (.00009*), HFD+MG_13 (.024*)	.108

Abbreviations: OD, optical density; PAS, periodic acid-Schiff; SD, standard deviation; SW, Shapiro-Wilk test for normality of distribution; B, Bartlett's test for homogeneity of variances; F, ANOVA test; T, Tukey's test for multiple comparisons of means; D, Dunnett's test for multiple comparisons of means with the CTRL group. *The test yields a statistically significant p-value ($p < 0.05$).

MUC2

Table S6. *Statistical comparisons of **proximal** CTFC $\times 10^{-4}$ values between controls (CTRL), high-fat diet (HFD), HFD+MG_13, and HFD+PI groups.*

**MUC2**	Mean ± SD	F (p-value)	SW (p-value)	B (p-value)	T (p-values)	D p-value
CTRL	22.13 ± 5.79	3.423 (.037*)	0.966 (.564)	5.929 (.115)	HFD (.780), HFD+MG_13 (.231), HFD+PI (.031*)	-
HFD	19.64 ± 5.52				CTRL (.780), HFD+MG_13 (.737), HFD+PI (.191)	.669
HFD+MG_13	16.95 ± 3.96				CTRL (.231), HFD (.737), HFD+PI (.716)	.151
HFD+PI	14.15 ± 1.78				CTRL (.031*), HFD (.191), HFD+MG_13 (.716)	.018*

Table S7. *Statistical comparisons of **distal** CTFC $\times 10^{-4}$ values between controls (CTRL), high-fat diet (HFD), HFD+MG_13, and HFD+PI groups.*

**MUC2**	Mean ± SD	F (p-value)	SW (p-value)	B (p-value)	T (p-values)	D p-value
CTRL	11.41 ± 1.17	44.29 (5.1e-09*)	0.944 (.203)	3.908 (.272)	HFD (.0004*), HFD+MG_13 (.0004*), HFD+PI (<.0001*)	-
HFD	7.55 ± 1.63				CTRL (.0004*), HFD+MG_13 (1.00), HFD+PI (<.0001*)	.0002*
HFD+MG_13	7.55 ± 1.64				CTRL (.0004*), HFD (1.00), HFD+PI (<.0001*)	.0002*
HFD+PI	2.54 ± 0.67				CTRL (<.0001*), HFD (<.0001*), HFD+MG_13 (<.0001*)	.0001*

Abbreviations: CTFC, corrected total cell fluorescence; SD, standard deviation; SW, Shapiro-Wilk test for normality of distribution; B, Bartlett's test for homogeneity of variances; F, ANOVA test; T, Tukey's test for multiple comparisons of means; D, Dunnett's test for multiple comparisons of means with the CTRL group. *The test yields a statistically significant p-value ($p < 0.05$).
